# Creating Art Together as a Transformative Process in Parent-Child Relations: The Therapeutic Aspects of the Joint Painting Procedure

**DOI:** 10.3389/fpsyg.2018.02154

**Published:** 2018-11-13

**Authors:** Tami Gavron, Ofra Mayseless

**Affiliations:** ^1^Art Therapy Department, Tel-Hai College, Tel-Hai, Israel; ^2^Graduate School of Art Therapy, University of Haifa, Haifa, Israel; ^3^Faculty of Education, University of Haifa, Haifa, Israel

**Keywords:** dyadic art psychotherapy, parent-child psychotherapy, joint painting, transformation in parent-child relations, parent-child relationship

## Abstract

The relationships between parents and children contain implicit aspects, which are non-conscious and non-verbal, in addition to explicit ones. Both explicit and implicit aspects are central to understanding the dyadic dynamics and are implicated in psychotherapy processes and outcomes. Visual symbolization has a unique value as a channel of expression that can capture the implicit characteristics of relationships. Creating art together goes even further because it allows the presence of implicit representations of the relations *in vivo*. These representations can then be transformed through the joint process of creation, which has a unique potential to unleash reflective capacities when it is experienced in a playful and safe context. This paper presents a qualitative study that is part of larger mixed-methods research with 87 mother-child dyads (with children 9 to 12 years old). Dyads were administered the Joint Painting Procedure (JPP), which includes dyadic painting by the parent and child on the same paper and is used for evaluation and treatment in the field of parent-child therapy and art therapy. The study’s objectives were to uncover and better understand the unique therapeutic aspects that such method allows and its potential to impact parent-child relationships. The findings of the qualitative study indicated that the JPP enabled several dynamic processes such as pleasure and fun, bi-directionality, mutual regulation, mentalization, and mutual recognition, which together created a salient positive transformation in the relationship. Through the JPP, a new transformative aspect of relations emerges and enables new and different modes of communication and interactions in about half of the dyads and a lesser and partial positive transformation in about a third of them.

## Introduction

Parent-child relationships are among the most important factors that contribute to children’s’ adjustment and well-being (Gilmore and Meersand, 2014; Koehn and Kerns, 2016; Wang and Fletcher, 2016). These relationships contain implicit aspects, which are non-conscious and non-verbal, in addition to explicit ones (Lyons-Ruth et al., 1998; Fonagy, 2001; Granot and Mayseless, 2001; Gavron and Mayseless, 2015). Both explicit and implicit aspects are central to understanding the dyadic dynamics and are implicated in psychotherapy processes and outcomes (Fonagy, 2001, 2015; Stern, 2004). Visual symbolization has unique value as a channel of expression that can capture and express the implicit characteristics of relationships (Madigan et al., 2003; Goldner and Scharf, 2011). Creating art together goes even further because it allows the presence of implicit representations of the relations *in vivo*. In addition, joint art activities enrich the individual and enhance a unique shared expression of every dyad, thus helping to create a distinctive dyadic narrative (Proulx, 2003; Gavron, 2011).

This paper explores and describes the therapeutic aspects of using art in parent-child psychotherapy, focusing on a unique and highly promising method of relationship assessment and intervention through painting – the Joint Painting Procedure (JPP). In the JPP, parents and children create art together following a planned and structured process. Although this method and others (e.g., Proulx, 2003; Landgarten, 2013) have been used clinically for quite some time and have demonstrated promising outcomes (e.g., Gavron and Mayseless, 2015), we currently have only a preliminary understanding of the process by which joint painting such as that done with the JPP affects the relationship during a therapeutic session. This paper presents the qualitative part of larger mixed-methods research that examined the impact of joint art creation on children’s adjustment. The goal of the qualitative study was to uncover and better understand the unique therapeutic aspects that such method allows and its potential to impact parent-child relationships. The focus of the larger study was mother-child relationships, though father-child relationships are equally important (Carlson et al., 2004). Many researchers point out that positive and secure relationships with mothers support child adjustment and well-being in various aspects (Gilmore and Meersand, 2014; Wang and Fletcher, 2016).

We first discuss the centrality of implicit aspects of relationships and the distinct nature of parent-child art psychotherapy model, and then we present the JPP as a central method within such therapeutic model and its clinical potential leading to the main focus of the present study.

### Implicit and Explicit Aspects of Relationships and Art Creation

Since the 1990s, researchers have begun to emphasize two aspects of human communication that occur simultaneously – the implicit and explicit aspects of a relationship. These two aspects evolve simultaneously in human communication over the years (Lyons-Ruth et al., 1998; Stern, 2004; Pally, 2005; Fosshage, 2011). Explicit communication develops from the second year of a child’s life, when children begin to use language for communication. The explicit aspects of the relationship are conscious, declared, and belong to the spoken language (Stern, 2004; Pally, 2005). The implicit aspects of relationships are connected to procedural and unconscious processes and can be expressed through a non-verbal manifestation such as art (Madigan et al., 2003; Goldner and Scharf, 2011). Implicit expression represents a central and essential aspect of relations in general and of parent-child relationships in particular (Stern, 2004; Pally, 2005; Schore, 2012). Art-based therapy provides a significant and established way to assess and attend to the implicit aspects of relationships, especially in parent-child relationships when children are not yet adept in articulating what they feel and think.

Focusing on art psychotherapy, Bucci (2011) emphasized that verbal language cannot contain all aspects of communication between people and underscored that we need to attend to three levels of communication: verbal, symbolic non-verbal, and subsymbolic (Bucci, 2011, 2014). The verbal level is related to language as communication and is expressed through a shared conversation during the therapeutic meeting. The non-verbal symbolic level of communication is expressed through metaphorical and visual images. The subsymbolic level of communication is related to expression through non-symbolized content, such as physical and sensory procedures, and through colors, lines, and shapes.

The processes of making art in parent-child art psychotherapy can simultaneously contain all three of these communication pathways (Markman Zinemanas, 2013; Gavron, 2015). But most importantly, the joint art-based expression during parent-child interaction in psychotherapy often uncovers a clear portrayal of the implicit dyadic interaction in motion. Through the dyad’s behavior and creative expression, one can observe and get access to a new understanding of the implicit aspects of the relationship (Mitchell, 2000; Stern, 2004). Mitchell (2000) and Stern (2004), both central figures in relational psychotherapy, even defined the implicit intersubjective and real-time encounter of the parent and the child as a central port of entry into the implicit representations of relationships and hence into the capacity to change them (Mitchell, 2000; Fonagy, 2001; Stern, 2004).

In fact, often only through attending to the implicit aspects of the relationship is deep and crucial understanding of the parent-child relationship revealed (Pally, 2005; Schore, 2012, 2014). Moreover, often these implicit aspects are the target of change, and with their transformation and their becoming more explicit, the quality of the relationship also transforms (Lyons-Ruth et al., 1998; Stern, 2004). Such therapeutic processes are the focus of parent-child art psychotherapy (Gavron, 2013, 2015).

### Parent-Child Art Psychotherapy

Parent-child art psychotherapy is a pioneering and innovative approach as part of the development of art therapy with children (Regev and Snir, 2014; Taylor Buck et al., 2014; Snir and Regev, 2018). It is a psychodynamic-developmental model that uses visual symbolization to express, communicate, and create change in the dyadic relationship. The creative art-based interventions of this model enable the evaluation and treatment of the dyad, focusing on the implicit aspects of the relationship (Gavron, 2011, 2015). The parent-child art psychotherapy model that is discussed in this study grew out of the principles of Haifa dyadic therapy, which is a clinical model for treating relationship disturbances during childhood (Harel et al., 2006; Kaplan et al., 2011). The Haifa model, which has a psychoanalytic-relational orientation, emphasizes the relationship between the parent and the child that is expressed and developed during the therapeutic encounter. It also emphasizes the developmental stage of the child, as a part of the child’s context and ecological system (Harel et al., 2006). Furthermore, the Haifa model focuses on the development of mentalization, which is an emotional-cognitive ability that helps to understand the self and the other in terms of mental states such as emotions, intentions, and wishes (Slade, 2005; Kaplan et al., 2011; Tessier et al., 2016).

These core characteristics of the Haifa dyadic model are also central in parent-child art psychotherapy. Yet parent-child art psychotherapy also intentionally prompts creative processes, using art materials within the art therapy room (Gavron, 2015). Creating art in parent-child art psychotherapy takes place in a number of ways: sometimes the child creates and the parent observes, sometimes the partners create alongside each other, and often the parent and child make an artwork together (Gavron, 2011; Snir and Regev, 2018). The visual symbolization in the parent-child encounter that comprises the artistic product and process enables a unique meeting space for communication and self-expression. While often parents communicate verbally and children express themselves through play, the artistic symbolization facilitates a unique way of being together. In particular, making art together as in the JPP, which is the focus of this study, invites a unique setting with great therapeutic potential. Using playfulness and imagination during joint art creation often leads to the formation of a unique narrative of the dyadic relationship and enables communication that is not often conveyed verbally (Proulx, 2003; Rubin, 2005; Gavron, 2015).

Despite the existence of clinical insights arising from joint art creation during parent-child art psychotherapy (Proulx, 2003; Wadeson, 2010), we currently do not understand what the exact dynamics of these processes are and how they evolve. Hence we lack a more explicit model of the interpersonal processes experienced during the therapeutic process and how they unfold and develop. This is the focus of the present study, which used the JPP to shed light on such processes.

### The JPP – Assessing Parent-Child Relationships

The JPP is an art-based assessment and clinical intervention that focuses on implicit aspects of the parent-child relationship. It comprises a structured five-step process in which both partners paint on the same paper, first working separately side by side and then painting together on a shared area of a single piece of paper. In the first step, the parent and child are asked to use a pencil to mark a personal space on a shared sheet of paper. Next, each partner paints inside his or her personal space using gouache or tempera paints. This is followed by an instruction to paint a frame around the painted space and then to paint a path from that frame to the frame painted by their partner. In the fifth and final step, the parent and child are asked to paint the rest of the paper together. After painting, the parent and child look at the painting with the therapist, discuss the shared experience, give the painting a title, and create a shared story about the painting. Following the administration of the JPP, the researcher or the clinician completes a structured protocol sheet that describes in detail every step of the dyadic procedure.

The JPP evolved from parent-child art psychotherapy, as well as from a long history of art-based assessments (Gantt and Tabone, 1998; Betts, 2006; Harel et al., 2006; Gavron, 2013; Schoch et al., 2017). The basic assumption of the analysis of the joint painting is that diagnostic information is embodied in the way in which the work is done, in addition to the symbolic content in the artwork. The emphasis is on how people paint and not just on what they paint (Gantt and Tabone, 1998). Indeed, the JPP analysis refers to the formal elements that exist in the joint painting, assuming that these elements give information about various implicit aspects of the relationship. At the same time, reference is made to symbolic content such as images and metaphors (Gavron, 2013; Gavron and Mayseless, 2015).

Many of the current art-based assessments are focused on individual painting and the internal representations of the painters (Betts, 2006). Even when drawing as a tool was used to assess relationships, such as in the use of family paintings, only the separate perspectives of the two partners were assessed (Madigan et al., 2003; Goldner and Scharf, 2011; Kim and Suh, 2013). Over the last two decades, extensive clinical literature has also discussed joint painting, as employed to understand family relationships (Kwiatkowska, 1978; Rubin, 2005; Wadeson, 2010; Landgarten, 2013). These joint painting tools contribute to the evaluation of the implicit dimensions of relationships *in vivo*, as they actually occur during an interactive, often therapeutic, session. Hence they provide a very important and significant way of understanding relationship dynamics. However, these tools are not yet often empirically tested (Betts, 2006).

The creation of the JPP reflects a formal and research-based consolidation of such clinical insights and hence provides access to an evidence-based, art-based tool for assessment and treatment (Gavron, 2013; Gavron and Mayseless, 2015). The JPP is accompanied by a validated manual that includes seven scales: individuation and autonomy, intrusion, mutual recognition, role confusion, motivation for relationships, emotional expression, and expression of implicit anger and aggression toward the other (Gavron, 2013, 2018). The manual describes the scales and includes descriptions of phenomena that characterize each level of every scale. The scales relate to the painting process and the final product, as well as to behavioral phenomena observed at each stage of the process (Gavron, 2013, 2018; Gavron and Mayseless, 2015). In order to transform a clinical art-based assessment (the JPP) into an evidence-based assessment tool and validate the manual, three steps were taken: (1) assessing inter-rater reliability between three judges who rated twenty dyads according to the JPP Manual, (2) examining the correlation between explicit aspects of relationships (from validated relationship questionnaires) and implicit aspects (from the JPP), and (3) predicting children’s adjustment based on the implicit aspects of relationships (as assessed by the JPP), beyond the prediction of explicit aspects of the relationships (as assessed by questionnaires and reported by mothers and children) and beyond the effects of the child’s temperament (Gavron, 2018).

In its original version, the JPP has mainly been used for evaluation of the therapeutic process at various points with the following clinical goals: (1) understanding the child’s internal world and relational representations within the context of interaction with the parent, (2) learning about the potential for growth and change in the dyad, as reflected in the continuous process of the joint painting, and (3) identifying and focusing treatment goals relevant to the dyadic relationships. As clinical observations have demonstrated the benevolent therapeutic value of the JPP, it has also started to be used as a clinical intervention tool (Gavron, 2015). Yet, we currently have only rich clinical experiences that attest to the unique and often benevolent dyadic process that occurs through the JPP and we lack a deeper understanding of the distinct aspects of this process and how it unfolds.

In what follows we present findings of the qualitative part of larger mixed-methods research. The objectives of the qualitative study were: (1) to understand and to shed light on the distinct dynamical process regarding parents and children during the JPP and (2) to apprehend how the JPP impacts and transforms the parent-child relationship and how such change evolves during the therapeutic session.

## Materials and Methods

### Participants and Their Recruitment

The participants included 87 mother-child dyads (43 boys and 44 girls ages nine to twelve) that underwent the JPP. In the larger mixed-methods study, mothers, children, and the children’s homeroom teachers also filled out questionnaires. The qualitative study presented here focused only on the JPP. Families were recruited from four public schools in the northern part of Israel and through social media. The researchers contacted the schools’ educational counselors, who distributed invitation letters describing the study. If the mothers were eligible (e.g., they had a child between the ages of nine to twelve) and willing, they were included in the research after signing a consent form. Written informed consent was obtained from the mothers both for their own participation in the research as well as for their children’s participation. The first author administered 58% of the JPP sessions. All the other sessions were administered by research assistants who learned the procedure from the first author. The JPP administrators wrote detailed description of the implicit and explicit aspects of the interaction (verbal, behavior and affective components) in each of the phases of the JPP administration with specific focus on exchanges, which are interactive such as, when A is doing something, B responds and A reacts to this response. Each phase of the procedure as well as the final product was photographed. The research was approved by the University of Haifa’s ethics committee.

To help the participants to feel more comfortable during the art-based process the administrators described the procedure and the use of materials, and suggested a short experience with the art materials before the actual procedure began. These measures along with the gradual progress of the JPP (i.e., moving from using a pencil to using paint and from individual painting into a joint one), created a sense of security and comfort among mothers and children. And indeed, none of the dyads expressed discomfort to engage in the art-making.

### Data Analysis

The analysis was based on the concept that meaning is created and understood within the context of social processes and that in order to understand different patterns in human relations, we need to look deeply at their behavior and expression during the JPP (Jeon, 2004; Starks and Brown Trinidad, 2007). The analysis of the qualitative data relied on narrative and phenomenological research perspectives (Betensky, 1995; Zilber et al., 2008; Kapitan, 2010; Spector-Mersel, 2010; Huss et al., 2012) to throw light on how the narrative of the relationship between the mother and the child developed throughout the JPP session. The research focused on the relationship narrative as it fluctuated and evolved (or not) *in vivo*. This method allowed analyzing the co-construction of a dyadic narrative of the relationship (Riessman and Speedy, 2007). Additionally, a phenomenological perspective was used in order to observe the art-based phenomena, which occurred through the process and in the product (Betensky, 1995; Bat Or, 2012; Huss et al., 2012). Such phenomenological perspective is based on composing formal elements and symbols that exist in the painting in order to understand the mother and the child experiences (Huss et al., 2012; Gavron, 2013).

The qualitative analysis of the relationship narrative included observation throughout the different phases of the JPP session of the following aspects: (1) verbal interaction and reflections, (2) implicit interaction through art-based phenomena (formal elements) which involved the way of creating, such as body gestures, pace, use of colors, shape, motion and textures, as well as themes and metaphors (Gavron, 2013), and (3) implicit and explicit behavior and implicit and explicit emotional affect of each partner and the dyad together which occurred throughout the process. These aspects were observed at the beginning of the JPP session, as well as throughout every structured phase of the procedure, analyzing the way these aspects evolved and changed in the interaction along the time, while focusing in particular on situations in which each partner responded to the other.

The qualitative analysis in this study was carried out in three stages: Stage 1 – narrative analysis of the relationship and how if at all, they evolve during each phase of the JPP based on all the sources (verbal-explicit, implicit-art-based and implicit∖explicit behavioral and affective expression). Thus the first stage included an in-depth look and analysis of the dyadic process focusing on the five stages of painting and the individual and shared phenomena that occurred at each stage of painting. In addition, the analysis focused on the behavior of the mother and the child during the various stages, the dynamics of the relationships, and the verbal interaction during and after the painting (i.e., conversation about the experience, naming and telling a story about the painting).

Stage 2 – creating a combined narrative of relationship throughout the whole JPP process for each dyad. The second stage thus involved analyzing all the data together in order to formulate insights for each dyad, in an attempt to integrate and understand the relationship dynamics, which included the process, the product, and the behavior.

Stage 3 – forming more general insights based on cross-case analyses (Seawright and Gerring, 2008). During this stage categories were formed and seven relational dynamic processes were identified. Our first two insights related to the observation that most of the dyads expressed pleasure and fun during the process and that most of the dyads went through a process of transformation. To better understand the process of transformation using cross-case analyses the authors delved into the data to uncover the other dynamic processes described in the findings section.

Although conceived as a linear process, it actually involves a cyclic one, within each stage as well as across the stages. For example, when a certain insight was reached at the third phase, a thorough check of specific dyadic cases was undertaken to verify the trustworthiness of such claim. During the qualitative analysis, a number of methods were implemented to strengthen the reliability, trustworthiness, and credibility of the analyses (Tracy, 2010). In the analyzing process a triangulation across sources of data was used in order to increase reliability (Shkedi, 2005). This included the observation of the verbal interaction between the dyads and with the JPP administrator, the implicit behavior, which involved the art-based expression and the dyadic product (the painting) and actual behavior during the process. To heighten the probability that the researcher would accurately perceive what happened in the dyadic processes, the researcher relied on self-reflection and bracketing, using a research diary in order to examine the analyses and perceptions as accurately as possible, trying to focus on what came out of the painting process itself rather than relying on preconceptions. Additionally, the researcher (first author) consulted with several research colleagues and clinicians who were not involved in the study’s administration and with the research supervisor (second author) at many junctures during the analysis. This occurred both during the formation of general narratives for each dyad and during the third stage of data analysis when cross-cases generalizations were formed. In such situations the persons with who the first author consulted read thoroughly each written narrative and observed every joint painting in order to form their own impression. Such processes led to in depth discussions of specific cases as well as the general insights from the whole cohort of dyads and allowed re-checking and sometimes reformulating the preliminary analysis and insights of the first author. Finally, throughout the process, written thick descriptions of the process and the product were used. These provide rich, elaborate and detailed description of the phenomena, which is examined in the research (Wertz et al., 2011). Such details are essential in order for other researchers to evaluate and examine the phenomena in each dyad and grasp the generalizations.

## Findings

The findings demonstrated that the JPP process enabled a unique expression of the complex implicit relationship between mother and child. The dynamics that occurred during the JPP pointed to a multifaceted and evolving interpersonal dyadic experience. In most cases, a transformation occurred in the relationship throughout the process, indicated by the participants and through analyzing the artistic product. About half of the dyads showed a full positive transformation process, and about a third showed a partial positive transformation process. Few dyads did not show any change in the quality of the relationship throughout the process. The findings further uncovered several dynamic processes that appeared to be connected to each other and evolved throughout the joint painting. Together these processes bring about transformation in the relationships. In addition most of these processes evolved at the implicit level of communication, though sometimes a verbal interaction joined in. To illustrate the major findings regarding the unique processes that emerged we first present three case studies that allow thick description of these processes.

### Case Study #1

**FIGURE 1:**
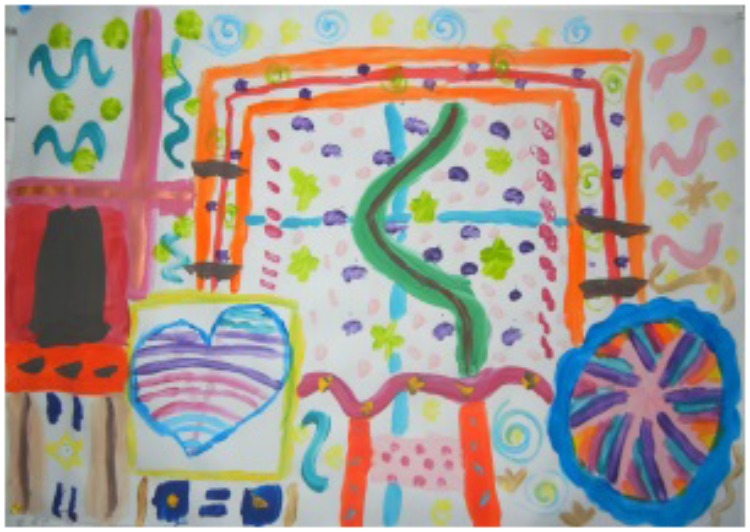
Joint painting by Gili and her mother (written informed consent was obtained from the creators for the publication of this image).

Gili (pseudonyms are used here), a ten-year-old girl, entered the art therapy room with her mother, very quietly with hesitant steps (Figure 1). She barely talked and avoided eye contact with the researcher. She started to paint the heart on the left side of the shared paper. She created various soft pastel colors by adding white to each color. She then added a yellow frame (with some added white color). Her mother painted a circle on the right side of the paper, starting with bright colors. At that point, the mother looked at Gili and pointed out that she was creating very special and soft colors. The mother asked Gili if she could borrow Gili’s soft pink color. Gili suddenly raised her head, smiled, looked at the researcher for the first time, and handed the pink to her mother. The mother used the pink in the center of her circle.

Later on, when Gili was asked to paint the path toward her mother’s space, she chose a bright orange color and painted an angular path on the upper part of the painting. Her mother painted the pink path between both shapes. When Gili observed her mother’s path, she said that her mom’s path was very beautiful and very rich and that her path was too long. Her mother pointed out that Gili’s path, although long, had the same very bright orange color she had used in her space, which she liked.

It seemed that Gili responded to her mother’s mentalized-based observation, as well as to the strong, pictorial existence of the mother’s path, and again something changed. In the next stage of the process, when Gili and her mother started to paint the shared part of the picture, Gili’s body energy and her mood changed dramatically. She painted with joy and laughter. Her mother responded to Gili’s change, and they both became involved in mutual play and creative dialog, where one would paint something and the other would respond through art. First, Gili used her pastel colors and her mother painted with the bright ones. They said that they played in turns, but each one in her favorite colors. Gili even mentioned that she mixed her unique colors. After a while, Gili began to use more bright colors and painted on large parts of the paper. At a certain point, Gili and her mother found themselves meeting with their two paintbrushes, both loaded with red paint, on the right side of the paper. They both burst into laughter and created the “red box.” Later on, while talking about the painting, they said that this was their special box, which no one could open except them. They decided to call the painting “Our Red Box.”

At the end of the session, Gili was talking freely and looked at the researcher and said that she really loved painting, especially creating new colors. Her mother said that she was very surprised and excited to see Gili so happy and free and mentioned that this was not common for her.

### Case Study #2

**FIGURE 2:**
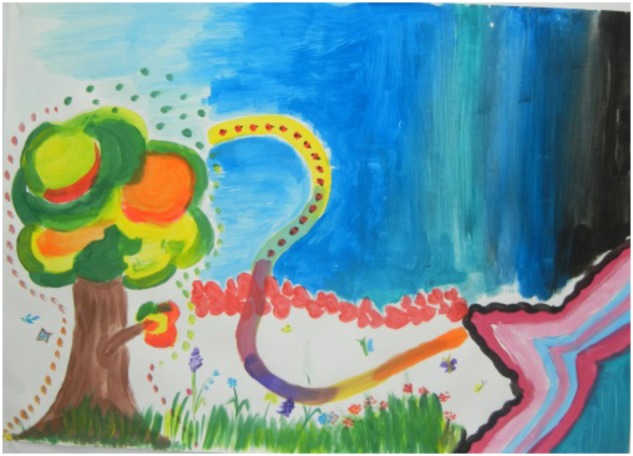
Joint painting by Tal and her mother (written informed consent was obtained from the creators for the publication this image).

Tal, a nine-year-old girl, and her mother entered the room with the mother coming in with a smile and Tal following her, looking displeased. When they began painting, the mother quickly marked her private space as the shape of a tree on the left (Figure 2). Tal watched for a few minutes and only then marked a shape on the right, which she called “a star.” The mother painted the tree with pleasure and freedom, using different colors. At the same time, Tal painted slowly and silently. She made sure to paint the lines with concentration, saying that the star was “half outside.” When asked to paint the path, the mother quickly painted a red flowery path. Tal watched and said angrily that she wanted to paint a long path between the two paintings and now she didn’t have enough space to do so. The mother said that they were each in a very different mood today. Tal then asked her mother if she could paint on her path. When her mother agreed, Tal painted a colorful path as she gently painted on part of her mother’s path. When both were invited to paint the joint part, Tal said she preferred to paint alone. She began to paint the black surface on the right upper side, while her mother painted two birds to the left of her tree.

Following that, the mother began to gently paint grass, flowers, and butterflies toward Tal’s star. Tal, who watched the mother, stopped painting the black shape and began to paint grass with flowers toward her mother. This appeared to be in response to the mother’s implicit expression. At this point, the atmosphere changed, and Tal painted freely while describing her painting. When they had finished painting the grass and the butterflies, Tal asked her mother if she could help her continue her painting on the upper part of the paper. The mother began to paint in light blue next to the tree, and Tal continued to paint in dark blue toward her mother. Both shades of blue met in the center of the paper. At the discussion following the painting process, Tal said that at first she did not want to paint with her mother. She went on to say that she discovered that it was fun and she felt happy to paint together. The mother said she was debating whether to make Tal come to the meeting, but she thought she might enjoy it. When both were asked to name the painting, Tal suggested “A Shared Country” and said: “It is a fun country; it’s a different country, like in the movies, where everyone can do whatever we want.”

### Case Study #3

Avi, an eleven-year-old boy, and his mother told the researcher that they had painted together many times before. They began painting and Avi very slowly painted blue waves on the left side of the paper. His mother painted in large brushstrokes on the other side. When asked to paint the frame, Avi painted an orange line around his personal space. His mother continued to paint her personal space, ignoring the request for a frame. They both painted two central paths. Following that, they painted the joint part without talking. The mother again painted with large brushstrokes in many colors, all over the paper. Avi moved to the other side and painted a few small gray dots, which took up very little space. Toward the end of the meeting, the mother painted a white frame around part of her personal painting. Only then, Avi painted few more dots in the center of the painting. During the shared observation, the mother said she enjoyed the process and wanted to name the painting “Fun of Two.” Avi did not want to give the painting a title. He said: “Well... I did not have so much fun... It was ok. I like the part with the dots; it’s much quieter there. The rest of the painting is too entangled with all those colors.”

It could be observed that there were major differences between the boy and his mother in various aspects: their pace and energy, their movements, and the space they each took on the paper, as well as the explicit description of their experience. When the mother took the space for expression, Avi moved to the side and was constrained in his implicit expression. Only when his mother framed part of her personal space and created a tangible separation did Avi feel safer to paint in the center. There was no evidence of them relating to each other’s actions or artistic expressions, and nothing actually was created together.

### Dynamic Processes

As exhibited in two of the case studies (#1 and #2), several major dynamic processes emerged during the JPP, and when all six of them were present, this led to transformation in the relationship. The third case portrayed a dynamic that did not lead to transformation. The dynamic processes are: (1) pleasure and fun, (2) mutual bidirectional effect, (3) development and evolvement of the relationship, (4) mutual regulation, (5) mentalization, (6) mutual recognition, and (7) transformation of the relationship.

#### Pleasure and Fun

Many of the mothers and children who participated in the study expressed a sense of pleasure and sometimes excitement from the process. Most of the mothers reported having a special experience with their children and a sense of closeness created during the experience that was not always available during daily life, while many children reported feeling good after the process. The expression of the “fun” feelings of the children and mothers related to the use of art materials and symbolic metaphors, which led to a creative process that enabled a new non-verbal authentic and playful expression. This could be seen, for example, in Gili’s saying that she most enjoyed mixing new colors. Playfulness and enjoyment also could be seen in the following example: Amit, a nine-year-old boy, painted a large and colorful flower in his personal space, while his mother painted her own flower with pastel colors. When asked to paint the paths, Amit stood up and painted a huge, thick brown path from the inside of his flower toward the upper part of his mother’s flower. “This is so much fun,” he shouted happily. When his mother added her pink path from underneath her flower to Amit’s flower, she said, “This is so beautiful.” Amit was laughing: “They [the paths] are hugging our flowers.” The joint procedure created a safe shared space for each partner where a different unique expression of each partner could evolve in a playful manner. As Tal said: “a fun country... where everyone can do whatever we want.”

#### Mutual Bidirectional Effect

There was a mutual bidirectional effect on each partner throughout the process, when one partner’s behavior affected the other and evoked change in their behavior, which in turn affected the first person. The non-verbal dynamics through painting created joint transactions of reciprocity and reactivity. For example, during the final stage of the process when painting together, Gili and her mother became involved in mutual play and creative dialog, where one would paint something and the other would respond. Another example is when Tal responded to the flowered grass painted toward her individual painting by painting her own grass toward her mother. In another example, ten-year-old Orr and her mother decided to paint blue sky in the joint area around their personal spaces. When the mother painted a white cloud, Orr responded with another cloud. Orr painted a bird and the mother painted a different kind of bird. They ended the process by painting a huge rainbow, when each took turns painting in a different color. In the case of Avi and his mother, the mutual bidirectional did not occur. They each seemed to be in their own space, not responding to or being affected by the other’s actions. It appeared that they ended the process without any change in their relationship.

#### Development of the Relationship – An Evolving Process

It seems that the processes of the joint painting allowed for the development of the relationship over time and in most cases led to a positive change in atmosphere and a sense of closeness. This was reflected, for example, in the change in Gili’s painting process from a hesitant experience to a playful and enjoyable game with her mother. Similarly, Tal was able to reconnect to her mother after she could explicitly express her ambivalent and angry feelings toward her. However, in a small percentage of the dyads this development did not evolve or evolved only partially. In Avi and his mother’s case, the boy said that he did not enjoy the process. More than that, although both partners were expressive, they did not engage in a process of receptivity and mutuality, and they seemed to work in a disconnected way. They did not experience the evolving process of the JPP, during which, as part of the bidirectional process and the development of the relationship, three major interrelated phenomena appear – mutual regulation, mentalization, and mutual recognition – enabling a transformation in the relationship.

#### Mutual Regulation

A process of mutual regulation occurred through the artistic action, when the parent regulated the child’s feelings and behaviors, and sometimes vice versa. Parents often closely observed the child’s sensory-emotional manifestations and identified, responded, and regulated those feelings – implicitly but sometimes also explicitly. For example, in Gili’s case, the vivid colors that the mother used seemed to affect and regulate Gili’s choice of colors, which later in the process became stronger and more expressive. Tal painting her black background, which turned into blue, seemed to be a response to the blue colors that her mother painted toward her. Another example was when ten-year-old Mike, who used his hands to cover most of their painting with messy mixed-brown colors, was asked by his mother to paint an actual image. When he chose to paint a house, he asked his mother to color inside after he made the outline of it. The mother painted the roof with her fingers, and Mike, excited that his mother was painting like him, painted the rest of the house with a paintbrush and created an image that he was proud of. It seems that the mother’s implicit response (using her fingers, but not her whole hand) created an empathic and organized regulatory experience that allowed Mike to express himself in a different manner than only being destructive. His mother, on the other hand, seemed to be influenced by Mike and painted more freely and playfully with her fingers. In Avi and his mother’s case, mutual regulation did not evolve and each partner retained his or her own pace, color, and rhythm.

#### Mentalization

Within the framework of the joint painting, verbal, emotional, and cognitive attributions relating to the behaviors, artmaking, or artistic product of the other took place. These attributions reflect a mentalization process, as they include understanding the other’s behavior as an expression of emotional and mental states and perceiving the self and the other as owning unique thoughts, feelings, and emotions (Slade, 2005; Verheugt-Pleiter et al., 2008; Bat Or, 2010). For example, in Gili and her mother’s painting process, the mentalization occurred when both of them could acknowledge and address the differences in their preferences and behaviors. At first they just exhibited the differences: Gili used gentle colors and her mother used brighter ones; Gili painted a long path, and her mother painted a short one. But later these enacted implicit processes were acknowledged and they could verbally reflect on them, leading to an experience of each of them knowing and accepting herself and the other and being known and accepted.

In the beginning of the process for Tal and her mother, both partners were different in their explicit and implicit expression. The mother’s painting was rich and composed of two images, which may have symbolized both of them. Tal, on the other hand, seemed to begin the painting process with ambivalence about being with her mother (defining the shape as “half outside” and creating the long path toward her mother). The shape of the star was almost outside and did not take up much space on the shared paper. In addition, it appeared that Tal expressed feelings of anger toward her mother through the sharp shapes and her verbal statements. When the mother verbally acknowledged the differences between them, she used mentalization and helped Tal to understand herself and feel understood. In fact, despite the length of the path, the last part that connected to the mother’s tree was made of colors and textures that resembled those of the mother on her personal space, which may indicate a desire to connect. It might be that the mentalization of the mother helped Tal to reconnect to her.

#### Mutual Recognition

The mutual regulation and the mentalization that occurred in most processes appeared to facilitate a process of mutual recognition, which comprises the ability to recognize the other as a subject with a separate inner world, while being in a mutual relationship (Benjamin, 1988, 2005; Gini et al., 2007). The mutual recognition took place in the joint painting and included simultaneous recognition of the self and the other as having a unique way of being and expressing but at the same time having the ability to create together out of a close and mutual relationship. Mutual recognition is different from mentalizing because it includes mutuality and the capacity to be both separate and connected. Furthermore it is more than mutual regulation in that there is a mutual recognition and acceptance of self, other, and the relationship. As can be seen in the case of Gili and her mother, the joint creative encounter led to the process of mutual recognition. The joint painting allowed for a unique personal expression in light of the other and at the same time a common representation evolved. Gili emphasized her unique way of expression through the colors, while acknowledging her mother’s separate and different manifestation. At the same time, both could share their colors, create an integrative game that showed on the paper, and paint a shared image that held a meaningful metaphor for their relationship.

During the process of Tal and her mother, the mother allowed Tal to express herself in an authentic way (to be on the side, to cover her path) and still expressed her desire to connect with her (through the tree image and the short and present path). It seemed that the transformation in Tal’s behavior and experience was made possible by two implicit factors, the first reflecting the mother’s recognition and the second reflecting the daughter’s. First, when Tal asked to go up on her mother’s path, her mother accepted it without criticism, thus allowing Tal to express ambivalence and perhaps anger. Second, when the mother painted the grass and the flowers toward Tal as a symbolic wish to connect, Tal responded by painting grass with flowers toward her mother and thus moved from a distant and angry state toward a desire to create together with closeness and pleasure. Tal’s lawn was similar to that of the mother; however, it had its own uniqueness. It seems that Tal responded to the mother’s non-verbal gestures and at the same time felt that she had a place to express herself authentically. Later on, Tal invited her mother to paint the upper part together. Similarly, the mother painted in the same style as Tal but in different colors. This shared painting enabled a unique expression of each person and at the same time led to a shared creative space. It also appears that Tal’s story, about the different country where everyone can do whatever they want, symbolically expressed her experience. It seems that the “shared country” represented the possibility of a unique shared space with her mother that would allow each of them an exclusive emotional experience, inner freedom, and yet a sense of closeness.

In another example, Harel, a ten-year-old, and his mother showed a partial profile of transformation when they failed to engage in mutual recognition. Harel and his mother seemed to enjoy the shared painting, using similar colors in their individual spaces. They responded to each other’s individual painting and shared some comment about similarities and differences. When they were asked to paint the paths, they painted similar paths that indicated two symbolic roads toward each other. However, when asked to paint the joint area together, Harel asked to cut his personal painting out of the paper and said that he wanted to end the session because he didn’t like painting together on the same paper. At his mother’s request, he agreed to paint a shared pool. Nevertheless, when the mother painted the pool, he covered it with a different color. When she painted a fish, he covered it and painted a shell, and that went on until the pool was all painted with Harel’s elements. The mother allowed the behavior and did not acknowledge it verbally. In this case, the dynamic process evolved up to a certain point with certain levels of mutual bidirectional effect, some mutual regulation, and little mentalization. However, these partners were not able to make room for individual, unique expression in the framework of a shared physical and emotional space, and mutual recognition did not develop.

The joint observation of the artwork at the end of the process also invites other processes of mutual recognition. In front of the joint artwork, each partner can find a sensory or visual-symbolic expression of his or her inner world, the inner world of the other, and the shared narrative of the dyad (Isserow, 2008). Gili and her mother made a story about a red box, which was private and belonged only to them. Inside the box one could find each individual’s unique expression, along with their shared dyadic being. The conversation that emerged from their joint observation expanded the possibility of mutual recognition. In this way, the joint observation of the child, the parent, and the therapist of the dyadic artistic product may serve as a container to the dyadic process. Furthermore, each partner can find that the other partner had her own unique thoughts and feelings about the process and the product, and such realization can support the capacity to relate to the other person’s point of view, along with the experience of closeness and mutuality. For example, Gili’s mother was surprised to hear that Gili’s most important experience was her ability to create new colors, which indicated her autonomous expression in the relationship.

#### Transformation of the Relationship

These processes often lead to transformation of the relationship, which enables the dyad to experience new ways to be with each other. This change is possible through the JPP creative process, which includes the sensory-symbolic encounter through art materials (e.g., mixing colors, shared motions) and symbolic images (e.g., the red box) in a state of creativity and play. During the JPP, implicit representations of relationships were enacted, met, and changed toward a transformative experience. The transformation of the representations could be seen through Tal’s images, which represented a transformative emotional process – from feeling resistant into being engaged and feeling free to express herself with positive and negative feelings. Her mother, at the same time, was also able to go through a transformative process – from being rejected into being supportive and helpful. The transformative process could also be seen when Gili and her mother experienced themselves as part of a new relational space – when a change could be seen in Gili’s behavior from being introverted to being a lively presence. Gili no longer experienced herself as remote and less good than her mother, but as being capable of unique creative expression that could be taken up by her mother and inspire her (e.g., when her mother used the soft pink color). The mother experienced herself as having the ability to communicate with Gili in a receptive manner, while helping her express herself in a safer manner.

Another example can be seen in the case of ten-year-old Ben and his mother. While Ben was painting over his mother’s images in red and orange colors in a destructive style, he said that he was painting a big fire. The mother reflected on the “fiery” way in which he painted and suggested that they together paint some wood for the fire. Ben was excited, and they added thick brown lines to the fire. As a result of the mother’s intervention, which contained regulation and mentalization at the same time, Ben was willing to create a controlled image of a fire and added an image of a boy sitting next to it. The mother said she loved the child and the fire and added an image of another child. It seems that throughout the joint painting, a transformation experience was created when the two partners experienced themselves in a new way. It seems that Ben was no longer experiencing himself only as tempestuous but as creative and as being appreciated by his mother. The mother may have been experiencing herself as having the ability to communicate with Ben in an accepting way, while helping him to be regulated.

### The Role of Verbalization in the JPP Process

Often during the JPP, it could be seen that explicit communication had an important role within the dyadic interaction. Of course, verbal communication is present among most parents and children in middle childhood as a means of cognitive and emotional expression (Gilmore and Meersand, 2014). But most important was the role of the verbal communication during the JPP as accompanying and highlighting the implicit evolving process of the dyad. The words supported the mutual regulation, e.g., the mother suggested to Ben that he paint an image out of the chaos. The words framed the mentalization process, e.g., the mother said to Ben that he is “fiery” today, and the words contained and gave structure to the transformation process. In another example, nine-year-old Judy concluded her session by saying to her mother, “Yesterday and today I was really angry with you, but I put it all on the paper, and I feel better. Now I can tell you why I was so angry.”

## Discussion

The findings of the current study uncover and facilitate better understanding of the unique therapeutic aspects that the JPP allows and its potential to impact parent-child relationships. The findings depict the dynamics of the implicit aspects of the parent-child relationships during the joint painting, which the study particularly focused on understanding. It appeared that the JPP served as a powerful intervention demonstrating the extraordinary potential for development and transformation in the parent-child relationship.

One of the main innovative discoveries of this research is the transformation process that actually occurs during the JPP. It is known that the JPP allows access to various characteristics in the parent-child relationship and supports implicit and explicit communication, as well as self- and shared expression (Gavron, 2013; Gavron and Mayseless, 2015). In this study, we realized that through the JPP, a new transformative aspect of relations emerges and enables new and different modes of communication and interactions. The joint creation during the JPP seems to invite an encounter different from the usual one in the everyday dyadic relationship. This special meeting expands the usual repertoire of communication and creates the transformative processes. This implicit and explicit dialog in motion leads to meaningful learning about new ways of being together (Tronick, 2003; Stern, 2004; Fonagy, 2015). The JPP creates positive reconstruction of various elements of the relationship. It is important to note that these changes occur even without the therapist’s intervention – through the continuous process of the joint creation and the implicit aspects of relations and the subsymbolic communication that this joint creation allows.

The ongoing mutually that evolves while creating together, construct implicit moments of meeting (Lyons-Ruth et al., 1998; Stern, 2004) that could lead to a change in the relationship. As seen in our findings, such a meeting can occur through different modes of communication (Bucci, 2014): through a common rhythm or movement while painting, through the meeting of shapes and colors, or through a shared image. Such a moment seemed to evolve when Gili and her mother accidently met with their paintbrushes loaded with a reddish paint. This was an implicit sensory meeting through color and touch, which apparently led to the painting of the container. It appeared that this container was a representation for their unique shared closeness in a synchronized way.

Another major finding of the study refers to processes or conditions needed in order for this transformation to evolve. Several such processes emerged as part of the JPP: (1) pleasure and fun, (2) mutual bidirectional effect, (3) development and evolvement of the relationship, (4) mutual regulation, (5) mentalization, and (6) mutual recognition. The context of pleasure and fun in the JPP appears to be significant. Play and creativity, which are voluntary actions for their own sake, enable pleasure, reward, and satisfaction (Schore and Marks-Tarlow, 2017). The playful art-based process allows for the presence of two points of view in a creative way at one point in time (Benau, 2009). Pleasure and fun are connected to Regev and Snir (2014)’s findings about the main facets in parent-child art therapy that facilitate positive feelings in the relationship. These pleasurable feelings create a common ground for the mental processes that follow.

Mutual bi-directionality occurs when both parent and child affect and are being affected by the other’s behavior (Harach and Kuczynski, 2005). The act of painting together provides a space for positive reciprocal exchanges and for implicit negotiations of matches and mismatches between them (Paschall and Mastergeorge, 2016). Harach and Kuczynski (2005) indicate that often such positive reciprocal exchanges occur in the context of play, which is similar to the fun and often joyful and autonomous process of the JPP. The reciprocal process entails some intimacy and companionship that often include shared laughter, shared pleasure and common understanding (Harach and Kuczynski, 2005). This co-creation therefore provides an opportunity to step out of the traditional hierarchical relations and engage in intimate implicit interactions that foster closeness and companionship. Together these experiences often lead to development and evolvement of the quality of the relationship during the JPP.

These processes often facilitate mutual regulation, which evolves through the sensory and tactile component of the shared art-based experience and has a mutual effect on both partners (Hinz, 2015). Tronick (2003) argues that mutual states of regulation teach the dyad how to be together in different contexts and support a synchronized state of co-creativity. Schore and Marks-Tarlow (2017) state that non-verbal symbolic representation, such as play and the arts, facilitates emotional regulation function because it arouses a variety of feelings that enact regulatory boundaries. The mentalization process occurs within the JPP in a non-verbal and sensory way, and at the same time the partners can verbally address the process (Bat Or, 2010; Bucci, 2011). The state of mutual recognition emerges as a continuation of the relational processes and enables expression of each of the individual in the dyad as well as shared expression, while being in a close and mutual relationship (Benjamin, 2005; Gini et al., 2007). The capacity to recognize the other as distinct from oneself and to respect the individuality and uniqueness of the other while retaining closeness and togetherness is a complex and highly rewarding dynamic in a relationship (Benjamin, 2005). Its creation and sustenance becomes a token of positive and benevolent relationship.

Most of the processes described here have already been discussed in the clinical and research-based literature as important facets of the parent-child relationship (Slade, 1999, 2005; Schore, 2014; Fonagy, 2015; Tessier et al., 2016; Schore and Marks-Tarlow, 2017). However, the current study uncovered that these processes are connected to each other and revealed how they evolve throughout the joint painting in order to create transformation in the relationship. It appears that most of the dynamic processes described in this study need to occur in order to create the transformation. One process may lead to another, and if one or more aspects are missing, such as the pleasure part or the bidirectionality effect, the transformation would probably not fully evolve.

Another important finding of this study indicates that the JPP contains both explicit and implicit aspects of communication at the same time and allows for various ways of being together, such as having an implicit experience and also having a shared discussion between the partners (Isserow, 2008; Taylor Buck and Havsteen-Franklin, 2013). As appearing in the interaction of the dyads, the implicit art-based processes often led to verbal discussions, which are important in parent-child communication in middle childhood (Gilmore and Meersand, 2014). The tangible and visual expression of the relationship in the product itself and in the process of producing it allowed parents and children to look at the representations that were created and to verbally discuss them (Bat Or, 2010). In this way, the explicit communication appears to integrate and articulate the implicit evolving processes (Bucci, 2014).

This study sheds light on how the creative encounter enabled by the JPP uncovers profound and often hidden dyadic processes between parents and children. Such knowledge of the intricate and often overlooked implicit aspects in the interaction can help clinicians, parents and researchers in their efforts to understand parent-child relationship in the therapeutic interactions. The findings of the study underscore the significance of the use of joint artmaking as an important tool in parent-child art psychotherapy and in parent-child psychotherapy.

### Limitation and Direction for Future Research

This study is based on a one-session meeting. Observation of the relationship dynamic as it is affected through creative art processes could benefit from a long-term art-based intervention study. Further, this study examined the relationship between mothers and children. It is important that future research will also address father-child implicit dynamics during co-creation. The JPP should also be examined with children in different developmental stages, such as young children and adolescents. Another limitation of this study was that the first author conducted the majority of the JPP sessions (58%) as well as the qualitative data analysis, which introduced a certain bias into the research. This limitation was partially overcome by depicting phenomena across the whole cohort of dyads including a large number of JPP sessions that were administered by others. Additionally, other fellow researchers, clinicians and in particular the second author also examined the data of the JPP sessions and evaluated the insights gained by them.

In sum, the study underscored the centrality of implicit aspects in parent-child relations and the great potential of joint art creation to elicit positive transformation in the relations. The study uncovered a variety of dynamic processes that occur during the joint art creation. These interconnected dynamic processes together create an interwoven choreography that can positively transform the quality of relationship.

## Ethics Statement

This study was carried out in accordance with the recommendations of the ethic comity in the Faculty of Social Welfare and Health Sciences at the University of Haifa. The protocol was approved by the faculty ethic comity. All subjects gave written informed consent in accordance with the Declaration of Helsinki.

## Author Contributions

All authors contributed to conception and design to the study and wrote the second draft of this manuscript. This study was made as a doctoral dissertation of TG. OM was the doctoral facilitator and organized with TG the database and the qualitative writing. TG preformed the analysis and wrote the first draft.

## Conflict of Interest Statement

The authors declare that the research was conducted in the absence of any commercial or financial relationships that could be construed as a potential conflict of interest.
